# A Gaussian Process State Space Model Fusion Physical Model and Residual Analysis for Fatigue Evaluation

**DOI:** 10.3390/s22072540

**Published:** 2022-03-25

**Authors:** Aijun Yin, Junlin Zhou, Tianyou Liang

**Affiliations:** 1 The State Key Laboratory of Mechanical Transmission, Chongqing University, Chongqing 400044, China; 20162488@cqu.edu.cn (J.Z.); 201807021017@cqu.edu.cn (T.L.); 2 College of Mechanical and Vehicle Engineering, Chongqing University, Chongqing 400044, China

**Keywords:** Gaussian process, state–space models, fusion driven model, fatigue evaluation, residual stress

## Abstract

Residual stress is closely related to the evolution process of the component fatigue state, but it can be affected by various sources. Conventional fatigue evaluation either focuses on the physical process, which is limited by the complexity of the physical process and the environment, or on monitored data to form a data-driven model, which lacks a relation to the degenerate process and is more sensitive to the quality of the data. This paper proposes a fusion-driven fatigue evaluation model based on the Gaussian process state–space model, which considers the importance of physical processes and the residuals. Through state–space theory, the probabilistic space evaluation results of the Gaussian process and linear physical model are used as the hidden state evaluation results and hidden state change observation function, respectively, to construct a complete Gaussian process state–space framework. Then, through the solution of a particle filter, the importance of the residual is inferred and the fatigue evaluation model is established. Fatigue tests on titanium alloy components were conducted to verify the effectiveness of the fatigue evaluation model. The results indicated that the proposed models could correct evaluation results that were far away from the input data and improve the stability of the prediction.

## 1. Introduction

With the ever-increasing demand for high efficiency, lightweight, and highly reliable equipment, challenges are put forward for study on fatigue evaluation and the characteristics of its key components [1,2]. During the service life of metal components, cyclic load often leads to fatigue damage; however, it is difficult to measure the fatigue state of components directly by sensors. At the same time, fatigue crack growth is deemed a stochastic process that is affected by uncertainties arising from various sources [3], such as intrinsic material properties, the environment, and so on [4,5,6]. Therefore, it is of great significance to study how to establish a fatigue evolution model.

The data-driven modeling method is a type of mathematical modeling, which uses process monitoring data to optimize the parameters in the mathematical model, in order to obtain a more accurate relation model. It has been widely used in complex physical processes and systems, such as intelligent detection [7], materials [8], medical health [9], etc. Generally, there are two types of data-driven modeling methods: one type includes artificial intelligence methods [10], such as neural networks, fuzzy algorithms, etc.; and the other type [11,12,13] includes the Gaussian process (GP), the Wiener process, hidden Markov, etc. The GP has been proven to be a powerful Bayesian nonparametric method for solving nonlinear regression or multi-class classification problems [14]. It is widely applied to establish fatigue evaluation models. Shenfang Yuan et al. [15] use a GP fatigue crack evaluation model to establish the mapping relationship between structural health monitoring signal features and the crack length and realized that the model dynamically updated. Mohanty et al. [16] proposed a Bayesian statistic-based multivariate GP for fatigue crack evaluation, which showed a strong non-linearity representation capability and also an improvement in accuracy by using the Bayesian method. However, in all this research, the GP models are all data-driven models using offline data, which is not directly related to the physical process.

The GP inherits the advantages of the Bayesian statistical method and kernel function method [17,18,19,20]. It can retain prior information from the physical model, and the number of hyperparameters of the model is small and has physical significance. Fully considering the advantages of the Gaussian process physical model fusion, the GP becomes part of the model. The GP can adapt to different physical processes through the kernel function when trying to expand dimensions and output the normal distribution. Through state–space models (SSM), the two different models can be fused. A SSM can fuse the GP and the physical model together to form a digital-model-driven method using a particle filter (PF), Kalman filter, or hidden Markov model, which have been used in battery health assessments [21]. A PF has been widely used to establish a fatigue evaluation and prognosis framework, for example, Yuan and Yang [22] realized the prognosis of crack propagation based on a PF and a Lamb wave, in which curve fitting was adopted to correlate the damage feature extracted from the Lamb wave signals to the crack length. Zhu et al. [23] proposed a probabilistic model for fatigue damage diagnosis and prognosis in which a GP surrogate model was established by employing the PF through integrating the physical model with field inspections while accounting for the associated uncertainties. Jian Chen et al. [24] combined the PF with crack prognosis, effectively mapping the feature of structural health monitoring signals to the crack length.

The fatigue state of components are often judged by crack detection based on the PZT sensor [15] or Eddy current sensor. In contrast, the residual stress is closely related to the evolution process of the component fatigue state, and residual stress is easy to measure. Residual stress modeling has the advantages of rapid detection and rapid evaluation, so it has a certain discussion value. Schwach et al. [25] showed that the residual compressive stress near the surface of the component could improve the rolling contact fatigue life, while the maximum residual compressive stress of the subsurface layer had no obvious effect on the fatigue life. Kodama et al. [26] proposed a logarithmic linear relationship model to characterize the evolution of residual stress relaxation under cyclic loading through fatigue tests. In addition to the current residual stress, Omar [27] believed that the fatigue cycle of the specimen was also closely related to the initial residual stress and the surface roughness of the component; hence, Omar proposed a residual stress evolution model for initial residual stress.

The aim of this work was to consider a data-physics model for the fatigue evaluation modeling problem of a linear physical process, such as residual stress. The details of component production and numerical measurement were largely uncontrollable, so they were regarded as random factors, including observation personality and product personality. The GP could output the probabilistic distribution results, and describe the uncertainty of the conclusion. Starting from the SSM, a GP was combined with the physical model through FP to establish a more accurate fatigue cycle assessment model.

The rest of the paper is organized as follows. Section 2 briefly introduces the traditional physical models. Then, a fatigue evaluation model is proposed based on the SSM that combined the GP and physical models. Through the solution of PF, the importance of the residual was inferred. Section 3 and Section 4 show the titanium alloy fatigue test to prove the effectiveness of the fusion-driven model, and compare it with the Kodama model and the GP. Finally, Section 5 concludes the paper.

## 2. Materials and Methods

### 2.1. Physical Model for Fatigue Life by Residual Stress

When the sum of applied load and residual stress is greater than the yield strength of the material, plastic deformation could occur, resulting in the relaxation and redistribution of residual stress [28,29,30]. The Kodama model [26] is the following linear logarithmic relationship, expressed as the relationship between the residual stress and the current cycle, as shown in Equation (1).

(1) $$\sigma_{N}^{rs}=A+mln\left(N\right)$$

Considering the impact of stress gradient, initial residual stress, material stress and strain, cold work hardening, and roughness, Omar [27] proposed an equation for the prediction of residual stress relaxation, as shown in Equation (2).

(2) $$\frac{\sigma_{N}^{rs}}{\sigma_{0}^{rs}}=A{\left(\frac{1}{C_{w}}\right)}^{m}{\left(N\right)}^{B}$$

where $\sigma_{N}^{rs}$ is the residual stress of the member after *N* loading cycles and $\sigma_{0}^{rs}$ is the initial residual stress. $C_{w}$ indicates the degree of cold work hardening. A,m are constants depending on the load strength and shot peening strength. B represents the coefficient of equation for the prediction of residual stress relaxation. Meanwhile, neglecting the difficult measure $C_{w}$ in Equation (2), Omar also proposed a modified model, Equation (3), under the premise of constant load,

(3) $$\frac{\sigma_{N}^{rs}}{\sigma_{0}^{rs}}=g{\left(N\right)}^{C}$$

where g is constant and C is the relaxation exponent, which is dependent on the applied load and shot-peen intensity.

### 2.2. Gaussian Process

A GP believes that the prediction results of the model and the training data conform to the joint Gaussian distribution, so the probability density function (pdf) of the prediction results can be obtained by solving the marginal density. Furthermore, a GP can make the model very flexible through the design of different kernel functions, and the appropriate kernel function can be selected according to the actual physical process. This method is widely used in mechanical performance evaluation, life prediction, financial stock trends, and other fields [31,32,33,34].

A GP assumes that any set of multivariate features follows the joint Gaussian distribution, so that the target conditional distribution is obtained by calculating the edge probability density of the joint distribution. Suppose that a dataset of a known evolutionary process for residual stress with fatigue cycle set (*X, Y*) consists of $X={\left[x_{1},\ldots ,x_{n}\right]}^{T}$, $Y={\left[y_{1},\ldots ,y_{n}\right]}^{T}$, where n is sample size. The joint Gaussian distribution of GP is shown in Equation (4).

(4) $$\left(\begin{array}{c} Y\\ f\left(X^{*}\right) \end{array}\right)\sim N\left(\left(\begin{array}{c} \mu \left(X\right)\\ \mu \left(X^{*}\right) \end{array}\right),\left(\begin{array}{c} K\left(X,X\right)+\varepsilon^{2}I\\ K\left(X^{*},X\right) \end{array}\begin{array}{c} K\left(X,X^{*}\right)\\ K\left(X^{*},X^{*}\right) \end{array}\right)\right)$$

where, $f\left(X^{*}\right)\;$ is the potential mapping function, $X^{*}={\left[x_{1}^{*},\ldots ,x_{s}^{*}\right]}^{T}$ is the vector corresponding to the state to be evaluated, K is the kernel function to define the inner product relationship of the sample space, which represents the covariance matrix defined by the users, and ε is the corresponding noise term. Based on the closed operation of conditioning Gaussian, the predictive distribution $P(f\left(X^{*}\right)|Y,X,X^{*})$ is also a Gaussian process. By calculating marginal probability density, the pdf of $f\left(X^{*}\right)$, the mean function $\mu \left(Y^{*}\right)$, and covariance matrix $\Sigma \left(Y^{*}\right)$ can be expressed as in Equations (5) and (6).

(5) $$\mu \left(Y^{*}\right)=K\left(X^{*},X\right)*{\left(K\left(X,X\right)+\varepsilon^{2}I\right)}^{-1}*\left(Y-\mu \left(X\right)\right)+\mu \left(X^{*}\right)$$

(6) $$\Sigma \left(Y^{*}\right)=K\left(X^{*},X^{*}\right)-K\left(X^{*},X\right)*\;{\left(K\left(X,X\right)+\varepsilon^{2}I\right)}^{-1}K\left(X,X^{*}\right)$$

Usually,$\;\mu \left(X\right)$ is set to zero [31,35].

### 2.3. Gaussian Process State–Space Model Integrating Physical Model and Residual

**Figure 1:**
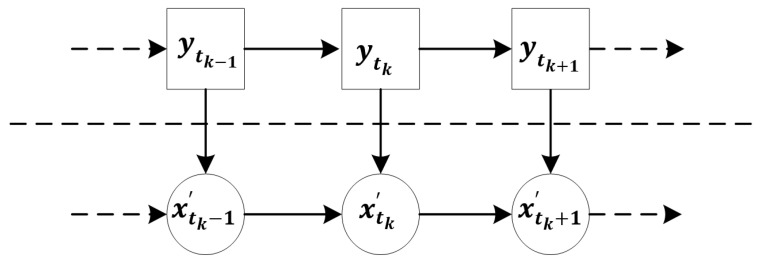
The SSM: $y_{t_{k}}$ is the hidden space state variable, $x_{t_{k}}^{\prime }$ is the observation space state variable.

Through the evolution of residual stress with each fatigue cycle, the state transfer equation of residual stress can be constructed as in Equation (7), which consists of a state equation and an observation equation, as shown in Figure 1.

(7) $$\left\{\begin{array}{c} y_{t_{k}}=f_{k}\left(x_{t_{k}},y_{t_{k-1}},x_{t_{k-1}},..y_{t_{0}},x_{t_{0}},v_{k-1}\right)\\ x_{t_{k}}^{\prime }=h_{k}\left(y_{t_{k}},u_{k}\right) \end{array}\right.$$

where $v_{k}$ is the noise in the hidden space, $u_{k}$ is the noise in the observation space, $f_{k}\left(\cdot \right)$ is the state transition function in the hidden space, and $h_{k}\left(\cdot \right)$ is the observation function.

The state transfer equation and observation transfer equation of the SSM need to be clear, but sometimes these functions are difficult to obtain directly. Either the state transition equation or the observation equation needs to be determined through the physical process. In addition, the mapping in the hidden space or the observation space may be inaccurate because of some sources of uncertainty, such as sensor placements. The goal of PF is to construct a set of N weighted particles ${\left\{y_{t_{k}}^{i},w_{t_{k}}^{i}\right\}}_{i=1}^{N}$ to represent the posterior pdf of the $y_{t_{k}}^{i}$ state particles at time k, as shown in Equation (8).

(8) $$p\left(y_{t_{k}}^{i}|x_{t_{1:k}}^{\prime }\right)=\overset{N}{\underset{i=1}{\sum }}{\hat{w}}_{t_{k}}^{i}\delta \left(y_{t_{k}}-y_{t_{k}}^{i}\right)$$

where N is the number of particles, ${\hat{w}}_{k}^{i}$ is the normalized weight of the *i*th particle at time k, δ is the Dirac function, and $y_{k}^{i}$ is the *i*th particle at time k. PF expresses the pdf of corresponding parameters through the weight of particles. A mean vector $E\left(\cdot \right)$ is used to describe the probability distribution, as shown in Equation (9).

(9) $$E\left(g\left(y_{t_{k}}\right)\right)=\overset{N}{\underset{i=1}{\sum }}{\hat{w}}_{t_{k}}^{i}g\left(y_{t_{k}}^{i}\right)$$

where $g\left(\cdot \right)$ denotes the distribution in the resample. Compare $E\left(\cdot \right)$ to $\mu \left(\cdot \right)$, the probability operations make PF and GP compatible. Hence, it is reasonable to use GP as the state transition function of PF, which represents a more complex physical process. In addition, the data-driven model can be considered by the SSM to form a data-physics fusion-driven model. The GP is taken as the state transition function of PF, as shown in Equation (10).

(10) $$\left\{\begin{array}{c} y_{t_{k}}=GP\left(x_{t_{k}},y_{t_{k-1}},x_{t_{k-1}},..y_{t_{0}},x_{t_{0}}\right)\\ x_{t_{k}}^{\prime }=A+m*y_{t_{k}} \end{array}\right.$$

where GP is the function of GP model. The observation function is the linear physical model. In the PF, $x_{t_{k}}$ and $y_{t_{k}}$ are the observation values. Through the GP model, the estimated $y_{t_{k}}$ could be obtained. Then, the physical model is used to obtain the estimated residual stress $x_{t_{k}}^{\prime }$. PF considers measured values and model estimates, so it means that the difference $\Delta x_{t_{k}}$ between $x_{t_{k}}^{\prime }$ and $x_{t_{k}}$ had a major impact on the results.

The importance of $\Delta x_{t_{k}}$ can be explained by follow the expression in Equation (10). The output of the GP model is the normal distribution, which makes the output of the linear physical model also a normal distribution, as in Equation (11).

(11) $$x_{t_{k}\;}^{\prime }\sim N\left(A+m*y_{t_{k}}^{\prime },m^{2}\sigma^{2}\right)$$

where $x_{t_{k}}^{\prime }$ and $y_{t_{k}}^{\prime }$ are from Equation (10) and N represents the normal distribution. For the state transfer equation, the two probability functions are most important, as in Equation (12).

(12) $$\left\{\begin{array}{c} y_{t_{k}}^{i}\sim P(y_{t_{k}}|y_{t_{k-1}}^{i})\\ w_{t_{k}}^{i}\sim P(x_{t_{k}}^{\prime }|y_{t_{k}}^{i}) \end{array}\right.$$

where $y_{k}^{i}$ are state particles and $w_{k}^{i}$ is the weight of each particle. Hence, Equation (10), following Equation (12), can be expressed as Equation (13).

(13) $$\left\{\begin{array}{c} y_{t_{k}}^{i}=y_{t_{k}}^{i}|x_{t_{k}}\sim \;N\left(f_{\mu }\left(x_{t_{k}}\right),f_{\Sigma }\left(x_{t_{k}}\right)\right)\\ w_{t_{k}}^{i}=x_{t_{k}}^{\prime }|y_{t_{k}}^{i}\sim N\left(A+m*y_{t_{k}},m^{2}y_{t_{k}}{}^{2}\right) \end{array}\right.$$

PF expresses the posterior probability distribution function through a set of N-weighted particles. The result regarding PF is $E\left(f\left(x_{t_{k}}^{i}\right)\right)$, the expectation of these weighted particles.

(14) $$\left(f\left(x_{t_{k}}^{i}\right)\right)=\frac{1}{N}\overset{N}{\underset{i=1}{\sum }}{\hat{w}}_{t_{k}}^{i}*f\left(x_{t_{k}}^{i}\right)\sim \int \left(x_{t_{k}}^{\prime }|y_{t_{k}}\right)f(y_{t_{k}}|x_{t_{k}})dx_{t_{k}}\;\sim \int k_{1}*e^{-\frac{{\left(x_{t_{k}}-A-m*y_{t_{k}}\right)}^{2}}{k_{2}}}*e^{-\frac{{\left(y_{t_{k}}-f_{\mu }\left(x_{t_{k}}\right)\right)}^{2}}{k_{3}}}dx_{t_{k}}\;\sim A*e^{-\frac{{\left|\left|x_{t_{k}}-A-m*f_{\mu }\left(x_{t_{k}}\right)\right|\right|}_{2}{}^{2}}{B}}=A*e^{-\frac{{\left|\left|x_{t_{k}}-x_{t_{k}}^{\prime }\right|\right|}_{2}{}^{2}}{B}}$$

where ${\hat{w}}_{k}^{i}$ is the normalized version of $w_{k}^{i}$, and *N* is the number of particles. $f\left(\cdot \right)$ is the state transition function. Following Equation (14), the difference ${\left|\left|\left|x_{t_{k}}-x_{t_{k}}^{\prime }\right|\right|\right|}_{2}{}^{2}$ between the measured value $x_{t_{k}}$ with the estimated value $x_{t_{k}}^{\prime }$ ($A+m*f_{\mu }\left(x_{t_{k}}\right)$) plays a key role in the GP-SS.

The essence of the Gaussian process state–space (GP-SS) model in this situation is to consider the difference between the input and the predicted result. The GP-SS model is further expressed by Equation (15).

(15) $$\left\{\begin{array}{c} y_{t_{k}}^{\prime }=GP\left(x_{t_{k}},y_{t_{k-1}},x_{t_{k-1}},..y_{t_{0}},x_{t_{0}},\alpha_{t_{k-1}}\right)\\ x_{t_{k}}^{\prime }=A+m*y_{t_{k}}^{\prime }\\ y_{t_{k}}^{*}=A^{\prime }+m^{\prime }*x_{t_{k}}\\ \Delta x_{t_{k}}=x_{t_{k}}-x_{t_{k}}^{\prime }\\ w_{t_{k}}=G\left(\Delta x_{t_{k}}\right)\\ y_{t_{k}}=\left(1-w_{t_{k}}\right)*y_{t_{k}}^{\prime }+w_{t_{k}}*y_{t_{k}}^{*} \end{array}\right.$$

where $G\left(\cdot \right)$ is the residual measurement function. The GP-SS model considers the importance of the residual, which has a major impact on the results.

### 2.4. GP-SS Model for Fatigue Evaluation

Combined with the assessment of the fatigue cycle by residual stress, the model could be corrected by the investigation of the residual stress. It made the model more accurate, relieved over fitting, and corrected deviated data. Another problem was how to use the residual stress in the fatigue cycle assessment. For the evaluation model, the method of measuring residual stress should meet some requirements: 1. The physical model cannot accurately evaluate the low cycle evolution, so the evaluation model should be inclined towards to the GP model in the low cycle; 2. The evaluation results of the GP model might have over fitting, so the filtering effect of the physical model for the GP model should be guaranteed in the high cycle, and the model should be inclined towards to the physical model; 3. If the output variance of the GP model is large, it should be inclined towards to the physical model. So the selected residual measurement method is an inverse sigmoid function, as in Equation (16).

(16) $$w=\frac{1}{1+e^{\frac{x-c}{b}}}$$

where w is the weight of the distance, b is the weight scale parameter and is equal to the value of GP evaluation variance after the physical model transformation, and c is the position parameter, where the mean value of residual stress is taken.

Meanwhile, the kernel’s selection of the GP has more evidence. According to the nature of the physical process, the kernel of these GP models is shown in Equation (17).

(17) $$\left\{\begin{array}{c} K=K_{linear}*K_{RBF}+K_{linear}\\ K_{linear}\left(x,y\right)=x^{T}\Sigma y\;\\ K_{RBF}\left(x,y\right)=\sigma^{2}*\exp\left(-\frac{|\left|x-y\right|{|}_{2}^{2}}{2*l^{2}}\right) \end{array}\right.$$

where $K_{linear}$ is the linear kernel, which expresses the linear relationship in Equation (1) through an inner product, and $K_{RBF}$ is the radial basis function  kernel, which expresses Equations (2) and (14) because the Omar model implies an exponential relationship between residual stress and the logarithmic cycle. K is used in both the GP model and the GP-SS model.

**Figure 2:**
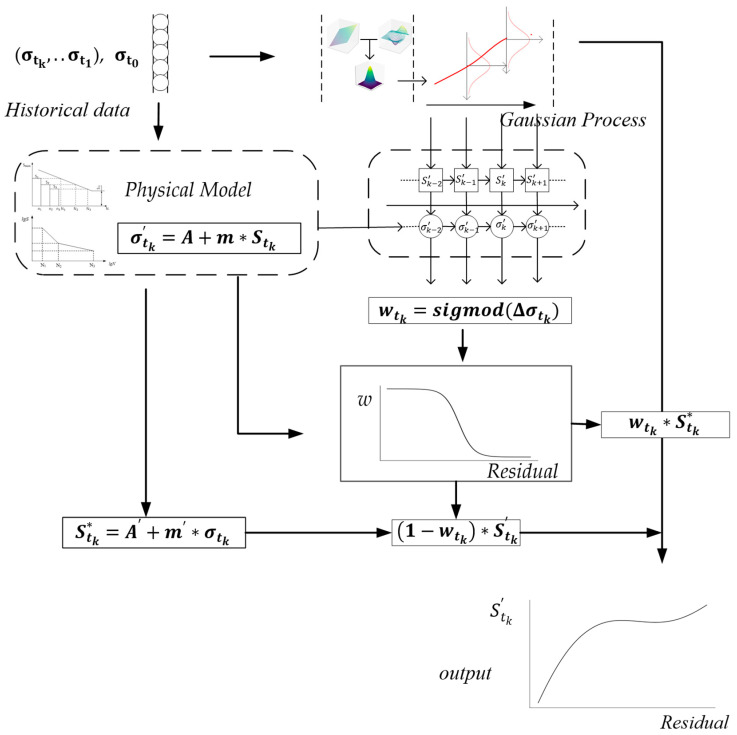
The GP-SS flow chart.

$\sigma_{k}=\left(\sigma_{t_{k}},\;\sigma_{t_{0}}\right)$ is used as the GP’s input, $S_{k}=S_{t_{k}}$ is used as a target, $\sigma_{t_{k}}$ is the current residual stress, $\sigma_{t_{0}}$ is the initial residual stress, and $S_{t_{k}}$ is the logarithmic cycle according to $\sigma_{t_{k}}$. Through this, the new model of fatigue evaluation of the metal components is proposed. The flow chart of the GP-SS with flow is shown in Figure 2.

In Figure 2, through the physical model and historical data, the model completes the trust assessment of the data-driven model.

The GP-SS model used the residual stress to modify the data-driven model away from the input dataset. The logarithmic residual stress ratio $\sigma_{k}$ was used in the GP firstly, then the GP could output the predicted logarithmic fatigue cycle. The residuals combined with the GP and the physical model were used to relieve over fitting and correct deviated data, as described in Section 2 and Section 3.

To sum up, the algorithm flow is shown in Algorithm 1.
**Algorithm 1:** The Algorithm of GP-SS**Input:** The residual stress ($\sigma_{t_{k}},..\sigma_{t_{1}}$), and the initial residual stress $\sigma_{t_{0}}$ **Output:** The logarithm cycle S.• Through the historical data and the current logarithm cycle ((($\sigma_{t_{k-1}},\sigma_{t_{0}}$),$\;S_{t_{k-1}}),..\left(\left(\sigma_{t_{1}},\sigma_{t_{0}}\right),\;S_{t_{1}}\right))$, optimize the GP model to obtain the predicted logarithmic fatigue cycle $\left(S_{t_{k}}^{\prime },..S_{t_{1}}^{\prime }\right).$ • The predicted residual stress $\left(\sigma_{t_{k-1}}^{\prime },..\sigma_{t_{1}}^{\prime }\right)$ is obtained by substituting the predicted logarithmic fatigue cycle $\left(S_{t_{k}}^{\prime },..S_{t_{1}}^{\prime }\right)$ into the physical model. Then, calculate the residuals $\left(\Delta \sigma_{t_{k-1}},..\Delta \sigma_{t_{1}}\right)$ to obtain the sequence of weight in Equation (15). • Through the physical model, the logarithmic cycle $\left(S_{t_{k-1}}^{*},..S_{t_{1}}^{*}\right)$, predicted by the physical model, is obtained. • Calculate the corrected assessment results $\left(1-w_{t_{*}}\right)*S_{t_{*}}^{\prime }+w_{t_{*}}*S_{t_{*}}^{*}.$

## 3. Experimental Setup

**Figure 3:**
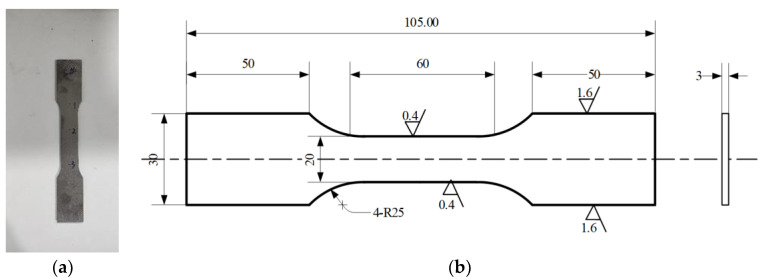
Shape design of specimen, which referred to GB/T 228.1-2010 *“Metallic materials—Tensile testing—Part 1: Method of test at room temperature”*: (**a**) specimen and (**b**) geometries of the specimen. All dimensions are in mm.

To prove the validity and advantages of GP-SS, the residual stress degradation tests were conducted on Ti-6Al-4V, Titanium-based alloy. The dog bone shaped components underwent shot-peened surface strengthening in the surface of the entire sample, as shown in Figure 3. The roughness was 0.4 μm. The shot-peening process is denoted by SP. The pellet diameter was 0.3 mm and the outlet pressure was 0.4 Mpa. Shot-peening time was 50 s and coverage was 100%.

**Figure 4:**
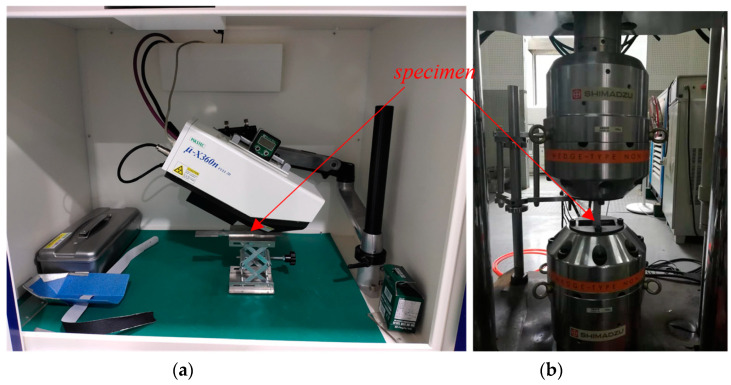
Experimental equipment: (**a**) μ-X360n X-ray diffractometer, residual stress measurement of specimens; (**b**) electro-hydraulic fatigue testing machine, tensile test of specimens. The titanium-based alloy specimens, the lower end of which is fixed on the clamping end and the upper end of which is fixed on the load end, was installed vertically on the fatigue testing machine.

We experimented on a component for the life cycle fatigue test, which was loaded cyclically to measure the residual stress in different cycles until the component broke. The five other components from the same shot-peening processes were only loaded until a specific proportion of the total life of the first component in order to measure the residual stress at that specific stage. The equipment and setup of the experiment is illustrated in Figure 4.

The measurement of residual stress by the μ-X360n X-ray diffractometer, based on the full technical method of two-dimensional detector, was a type of non-destructive testing, the measurement standard of which can be found in GB/T 7704-2008 “*Non-destructive testing-Practice for residual stress measurement by X-ray*”. Before doing the fatigue test, the residual stress of the titanium alloy introduced by the shot peening needed to be tested. Table 1 is the initial residual stress of each specimen.

Titanium-based alloy is a material with great plasticity. After one load, the titanium-based alloy underwent a great plastic deformation, with an elongation of about 18mm. In the subsequent loading cycle, it maintained its plasticity until it broke. In these tests, the load was 30 KN. The load stress ratio was 0.1, the loading frequency was 20 HZ, the load waveform was a sine wave, and cyclic loading was adopted. The measurement results are shown in Table 2 and Table 3.

## 4. Results and Discussion

The cost of conforming to the metal fatigue test was relatively high, so the total amount of data was small. It was not enough to control the total amount of data required by the model through the input dimensions. Therefore, an assumption was made that the collected data met a normal distribution, the mean of which was the measured value and the standard deviation of which was one third of the measurement tolerance. Through the whole life experiment, and the first stage’s experimental data, the model was established. Combined with the Monte Carlo method, the initial residual stress and the current residual stress were randomly sampled to increase the total number of samples.

**Figure 5:**
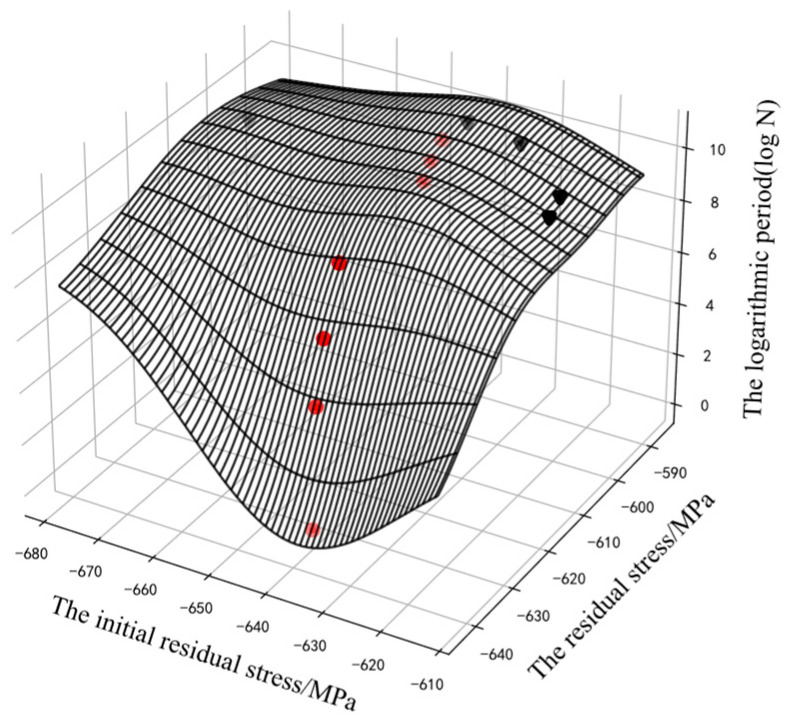
The assessment results of the GP.

**Figure 6:**
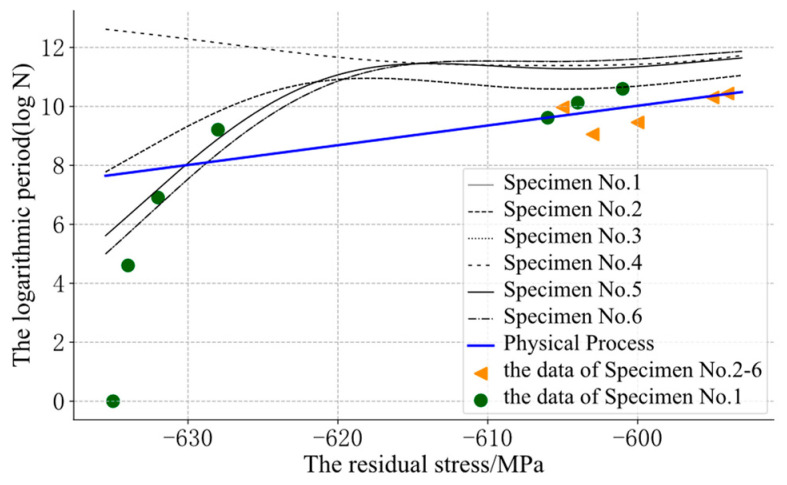
The assessment results of the GP: different line styles represent the fatigue relaxation process of different specimens. The triangle marks represent the data of specimens No. 2–6 and the circular mark represents the data of specimen No. 1.

The GP could use the residual stress and the initial residual stress to predict the logarithmic cycle. However, at points that were far away from the input data, the curve might appear to be unreasonable, such as in the reverse relaxation situation, as shown in Figure 5 and Figure 6.

For the fatigue evolution process of metal components, the residual compressive stress can effectively improve the fatigue strength of components, and it will relax with an increase in the fatigue cycle. Therefore, the residual stress was monotonous with the cycle. In Figure 6, some curves of the GP were obviously unreasonable. The curve of specimen No. 4 showed an opposite trend to the other curves, because its initial residual stress was far from that of the other samples. The prior knowledge about “monotony” could not be integrated into the model. The model could not reasonably predict the position far away from the input data and could not guarantee its monotonicity.

**Figure 7:**
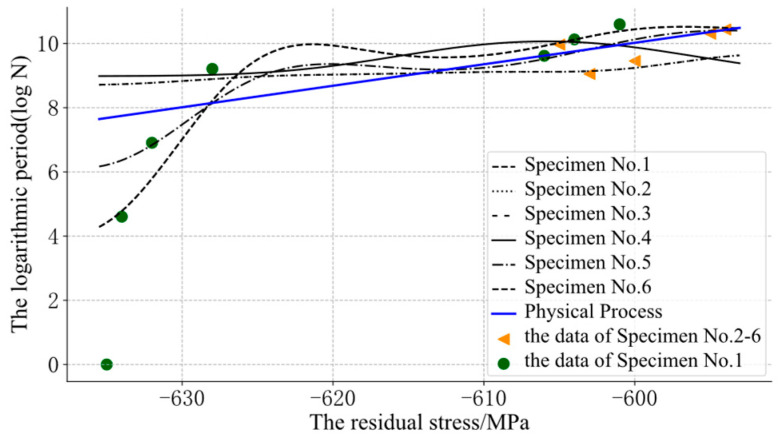
The assessment results of the SVR.

The support vector regression (SVR) model was added for the performance comparison, as shown in Figure 7.

**Figure 8:**
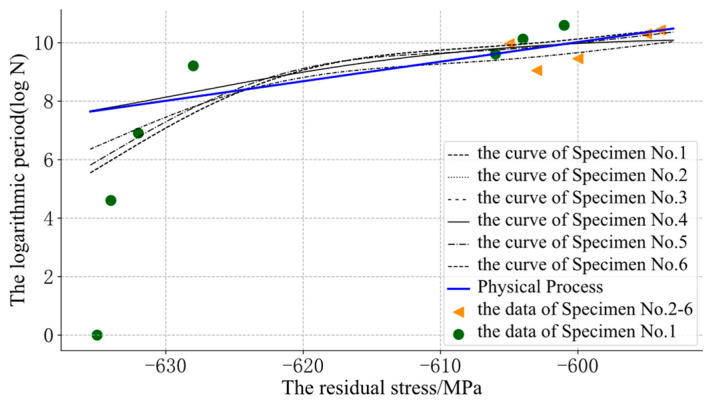
The assessment results of the GP-SS.

In Figure 7, some of the SVR was also obviously unreasonable and confusing because of the lack of prior information about the physical process. Through the GP-SS models, some curves could be optimized, as shown in Figure 8.

Figure 8 shows that the fatigue cycle evolution with the residual stress according to different initial residual stresses could be distinguished by residual stress differences. When the initial residual stress of the predicted position was far away from the initial residual stress of the training data, it was easy to distinguish the fatigue process curves corresponding to different samples in the same batch by introducing stress residuals; hence, we could obtain a better evaluation effect for each sample. Meanwhile, the difference obtained by the Kodama model gave the curve more monotony.

**Figure 9:**
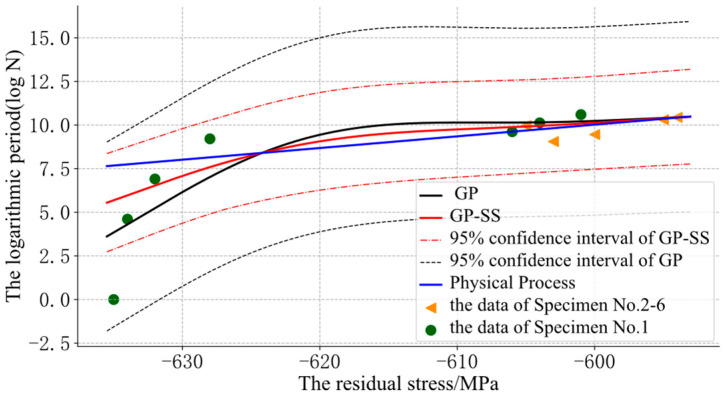
The results of different models for specimen No.1.

In Figure 9, the dotted line represents the 95% confidence interval in the GP and GP-SS models. The solid line represents the mean value predicted by different models. Compared with SVR and linear fit, GP could give a confidence interval, but this confidence interval had problems. This was because the confidence interval of the GP was affected by the adjacent data (on the X axis). Therefore, in the low cycle, due to the rapid fatigue relaxation stage, the adjacent data affected the determination of the confidence interval. GP-SS reduced the impact of the low-cycle fast slack data on the confidence interval of evaluation, making the confidence interval more accurate and forecasts more stable.

Finally, the evaluation error of each model was evaluated by Equation (1).

(18) $$error=\frac{\left|S_{predict}-S_{real}\right|}{S_{fatigue\_life}}*100\%$$

where $S_{predict}$ is the model evaluation result, and $S_{real}$ is the actual fatigue cycle of sample.

**Figure 10:**
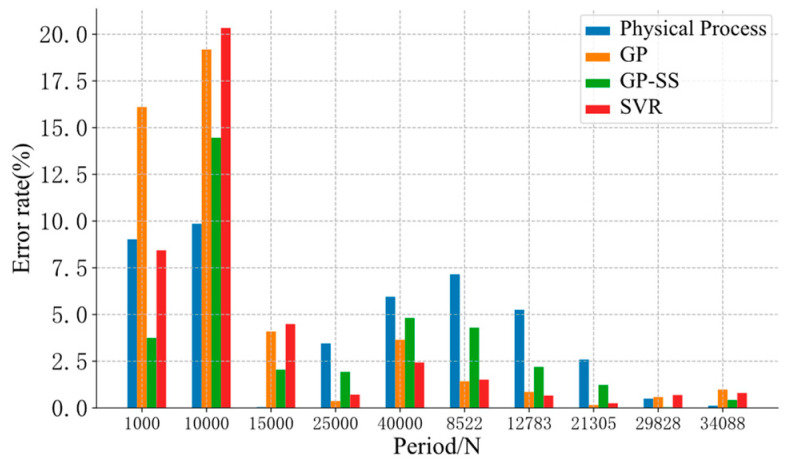
The error rate of different models.

The evaluation error based on the Kodama model is shown in Figure 10.

In Figure 10, at the data points of the phase experiment (period 1000, period 10000, period 15000, period 29828, period 34088), GP-SS improved the evaluation performance of GP. Although GP also had more accuracy in a part of data, its fatigue evaluation was uncontrollable when the data were far from the initial residual stress of the training data. An increase in residual stress increased trust in the physical models, alleviated the monotonicity problem of GP, and improved the performance of the GP-SS models in the middle stage. So, the GP-SS models could help to correct and stabilize the evaluation results.

## 5. Conclusions

This paper proposed a new model for assessing the fatigue cycle of metal components by residuals. Firstly, the initial residual stress and the probabilistic particles of the measurement results were introduced to solve the product personality and the observation personality. Unlike existing work, the fusion of a physical model and a data-driven model was realized through the SSM, and a residual-based fatigue evaluation model was proposed. Then, through the solution of the particle filter, the importance of the residuals was inferred and the fatigue evaluation model was established. Finally, the process and validity of the model were illustrated by the fatigue test of titanium alloy. In the test, the evaluation of the GP-SS models obtained probabilistic distribution results to describe the uncertainty of the conclusion. At the same time, the prior knowledge of the physical process corrected the evaluation results that were far away from the input data and improved the stability of prediction.

The proposed models could achieve remarkable evaluation performances in the middle stage of fatigue. The findings of this paper could be adapted to perform quality or fatigue evaluation and predict other linear physical processes. It should be noted that the model in some situations could not keep the monotonicity and it was unable to give the boundary conditions adaptively, which limited the application of the model. In future work, efforts will be focused on proposing some adaptive boundary determination methods. In addition to residual stress, the fatigue life of metal components is related to other physical factors, such as roughness. How to integrate these factors into the model is also one of the key ways to improve its evaluation effect.

## Figures and Tables

**Table 1:** The initial residual stress of specimens.

Specimen Number	Initial Residual Stress/MPa	Specimen Number	Initial Residual Stress/MPa
1	−640	4	−672
2	−620	5	−630
3	−620	6	−640

**Table 2:** The residual stress of specimen No. 1; specimen No. 1 was taken down after loading to a specific cycle, and the residual stress was measured. This operation was repeated until the specimen broke.

Load Cycle/N	Residual Stress/MPa	Load Cycle/N	Residual Stress/MPa
1	−635	10,000	−628
10	−634	15,000	−606
100	−632	25,000	−604
1000	−628	40,000	−601

**Table 3:** The residual stress of specimen No. 2–6; specimen No. 2–6 were removed at a specific ratio of the total loading period of specimen No. 1, and the fatigue cycle was measured, which was operated only once.

Specimen Number	Load Cycle/N	Residual Stress/MPa	Specimen Number	Load Cycle/N	Residual Stress/MPa
2	8522	−603	5	29,828	−595
3	12,783	−600	6	34,088	−594
4	21,305	−605			

## Data Availability

Not applicable.

## Abbreviations

**Symbols**	
GP	Gaussian process
SSM	State–space model
PF	Particle filter
pdf	Probability density function
GP-SS	Gaussian process state–space
SVR	Support vector regression
**Nomenclature**	
$\sigma_{0}^{rs},\sigma_{N}^{rs}$	Initial residual stress and residual stress
$A,m,A^{\prime },m^{\prime }$	Material constants in the physical model for fatigue life
$C_{w}$	Degree of cold work hardening
N	Number of cycles
B,C	Relaxation exponent
$x_{t_{k}},y_{t_{k}},x_{t_{k}}^{\prime },y_{t_{k}}^{\prime }$	State variable in the hidden or observation space
$\;\sigma_{t_{0}},\sigma_{t_{k}}$	Initial residual stress and residual stress
$S_{t_{k}},S_{t_{k}}^{*}$	Logarithmic cycle period
$w_{t_{k}}$	Weight of distance
$\Delta x_{t_{k}}$	The residual
$G\left(\cdot \right)$	Residual measurement function
$K_{linear}$	Linear kernel
$K_{RBF}$	Radial basis function kernel
